# Prolonged Responses to Cemiplimab in Advanced Cutaneous Squamous Cell Carcinoma: A Case Series

**DOI:** 10.7759/cureus.92346

**Published:** 2025-09-15

**Authors:** Alexandra P Guedes, Catarina L Fernandes, Inês B Freitas, Paula C Ferreira, Maria I Vilas Boas

**Affiliations:** 1 Oncology, Gaia/Espinho Local Health Unit, Porto, PRT; 2 Oncology, Matosinhos Local Health Unit, Porto, PRT; 3 Oncology, Instituto Português de Oncologia do Porto, Porto, PRT

**Keywords:** cancer-immunotherapy, cemiplimab, immune check-point inhibitors, non-melanoma skin cancers, squamous cell carcinoma of the skin

## Abstract

Cutaneous squamous cell carcinoma (CSCC) is a common skin malignancy with a subset of cases presenting as locally advanced or metastatic disease, often in elderly or comorbid patients. Cemiplimab, an anti-PD-1 antibody, has emerged as a standard treatment for advanced CSCC. This case series presents real-world outcomes of four patients (aged 75 to 83) with advanced CSCC treated with cemiplimab. Two patients had previous disease progression on first-line chemotherapy treatment. All four achieved complete responses, with time to response ranging from 6 to 24 months. Immune-related adverse events occurred in two cases, but were effectively managed. Cemiplimab was discontinued in all patients after sustained responses. Only one patient presented with a local relapse within 2 years after stopping treatment, after which cemiplimab was rechallenged with subsequent disease control. This case series highlights the significant and lasting responses that can be achieved with cemiplimab in advanced CSCC, even in older patients with comorbidities or prior treatment failure. Sustained disease control after treatment discontinuation favors time-limited immunotherapy and, in selected patients, cemiplimab rechallenge may represent a viable therapeutic option upon relapse.

## Introduction

Cutaneous squamous cell carcinoma (CSCC) is the second most common skin cancer, accounting for 20% of non-melanoma skin cancer cases, and its incidence is increasing globally [1-3]. Even though more than 90% of cases are diagnosed at early stages and are candidates for local treatment, locally advanced or metastatic CSCC carries high morbidity and a poor prognosis [4]. In this setting, options include mainly checkpoint inhibitor immunotherapy and platinum- and taxane-based chemotherapy (ChT). Epidermal growth factor receptor (EGFR) inhibitors (cetuximab) may have therapeutic activity in this context, but are not EMA-approved for CSCC [5].

Besides UV-light exposure and pre-malignant skin lesions, one of the main risk factors for CSCC incidence and progression is immunosuppression [6,7]. CSCC, among other skin cancers, usually carries a high Tumor Mutational Burden (TMB), approximately 50 mutations per megabase DNA, which represents increased neoantigen expression and is a predictive biomarker of response to immunotherapy [8]. These features make CSCC patients strong candidates for immune checkpoint inhibitor (ICI) therapy.

Cemiplimab, a human monoclonal antibody targeting the programmed cell death protein-1 (PD-1), has demonstrated significant antitumor activity with a favorable safety profile, leading to its EMA approval in 2019 for patients with unresectable locoregional or metastatic CSCC. This approval was based on the results from the nonrandomized phase 2 study EMPOWER-CSCC-1, which enrolled predominantly elderly pretreated patients and demonstrated an overall response rate (ORR) of 46% and a disease control rate (DCR) of 73% [9, 10]. The PD-1 inhibitor pembrolizumab has also shown meaningful and durable responses in advanced CSCC, as reported in the nonrandomized phase II trial KEYNOTE-629 [11]. While both agents share a similar mechanism of action, Cemiplimab is the only EMA-approved PD-1 inhibitor in advanced CSCC.

Despite the established efficacy of ICI, the characteristics and mechanisms underlying long-term responders remain poorly understood. This case series aims to explore the clinical outcomes and treatment experiences of four patients with advanced CSCC who had prolonged responses to cemiplimab therapy.

## Case presentation

Case one

**Figure 1:**
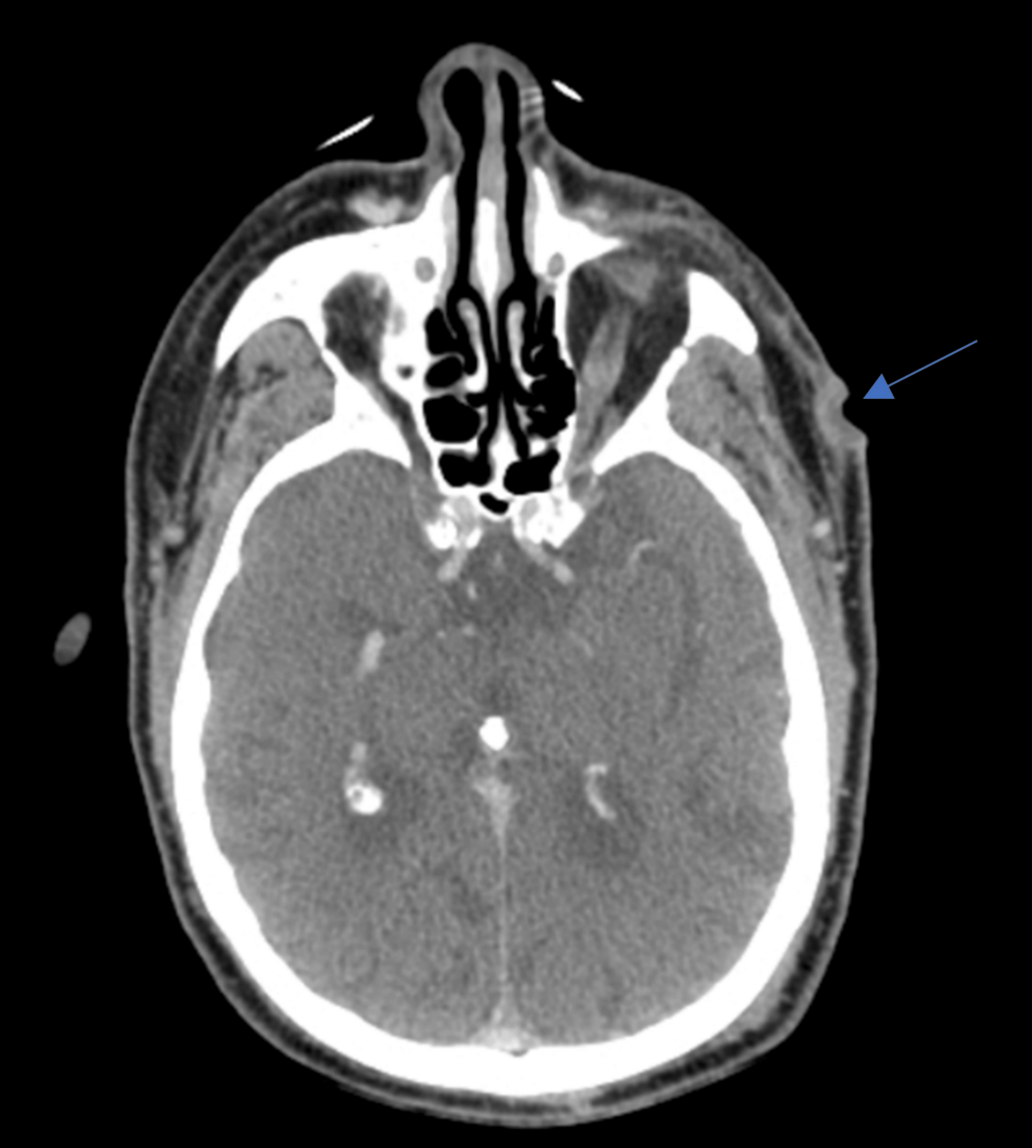
Staging CT scan. CT scan from March 2021 revealing a left malar lesion with cutaneous and subcutaneous thickening and ulceration. No distant metastasis was seen.

An 83-year-old male with a history of metabolic syndrome, hypertensive cardiopathy with diastolic dysfunction, stage III chronic kidney disease, and alcoholic cirrhosis had a 1.5 cm left temporal CSCC excised in July 2020. Due to deep margin involvement, post-operative radiotherapy was performed until October 2020 (60 Gy total). In March 2021, the patient presented with extensive local relapse. His Eastern Cooperative Oncology Group (ECOG) Performance Status was 1, and on the physical exam, two ulcerated lesions on the left temporal and malar region could be seen, 5 × 4 and 4 × 3 cm wide, respectively. Staging computed tomography (CT) scan revealed no distant metastases, but the lesions were deemed unresectable (Figure 1).

**Figure 2:**
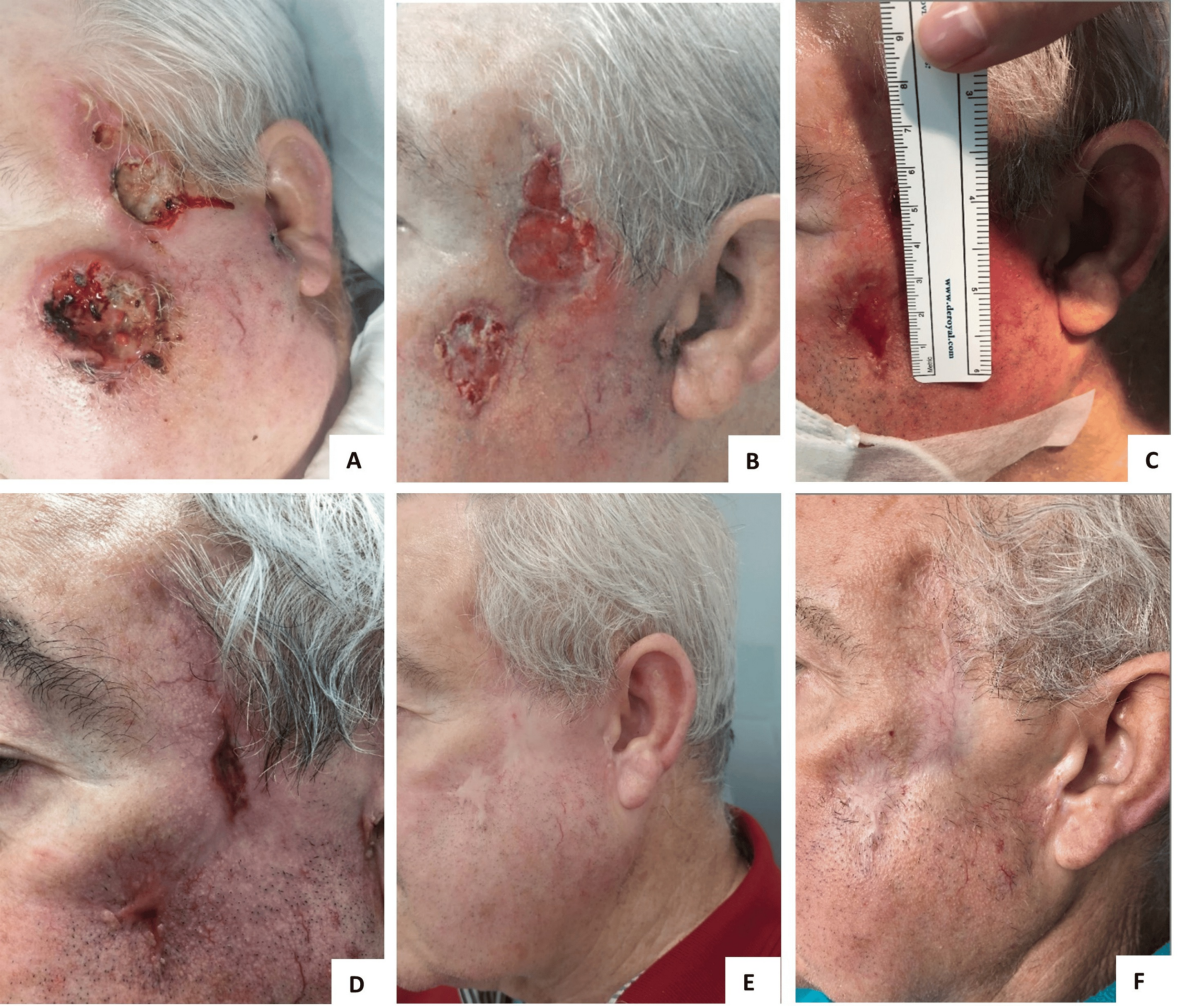
Response to cemiplimab in patient one. A) First clinical evaluation (March 2021) revealed two ulcerated lesions on the left temporal and malar region, around 5 × 4 and 4 × 3 cm wide, respectively. B, C, and D) Photographic documentation 2 months (July 2021), 3 months (August 2021), and 8 months (January 2022) after starting cemiplimab treatment, showing a rapid and meaningful clinical response. E and F) Sustained complete response 24 and 30 months after starting cemiplimab treatment (June and December 2023).

The patient started first-line palliative treatment with cemiplimab (350 mg administered intravenously every 3 weeks) in June 2021, having a meaningful clinical response to treatment (Figure 2). 

Imaging evaluation in March 2022 (after 9 months of treatment) revealed no evidence of disease. In September 2023, the patient reported diarrhea and rectal bleeding. A colonoscopy was performed, where distal colonic erythema could be seen, with loss of vascular pattern and friability. Histopathological analysis revealed a mixed inflammatory infiltrate, and cytomegalovirus testing was negative. A grade 2 CTCAE (Common Terminology Criteria for Adverse Events) immune-related colitis was diagnosed, which resolved after corticosteroid therapy and treatment discontinuation. At the time of article writing, the patient still had a sustained complete response 19 months after stopping treatment.

Case two

**Figure 3:**
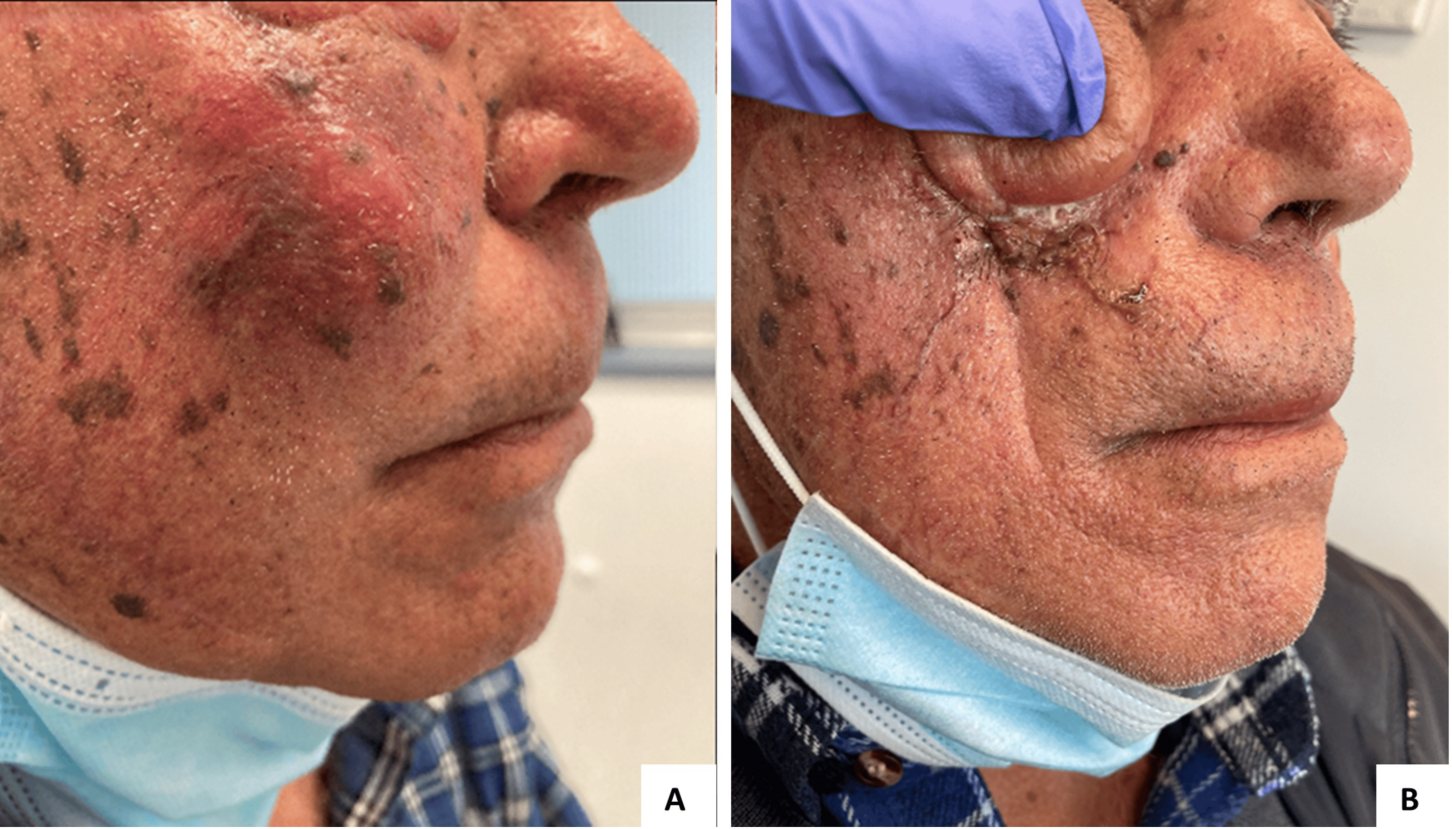
Response to cemiplimab in patient two. A) First clinical presentation (September 2020) revealed a 3x4 cm left malar lesion. B) Photographic documentation 6 months after starting cemiplimab (January 2022) treatment, showing evident clinical response.

An 82-year-old male with a history of benign prostatic hyperplasia and arterial hypertension had a deep 3 × 4 cm right malar lesion with progressive growth over the course of the previous 6 months, extending to the right lower eyelid. The patient’s ECOG-PS was 0, and surgery was performed in November 2020, which included a cervicofacial flap. Histopathology revealed grade 3 CSCC with 5 mm dermal invasion, and the deep margin was involved, but post-operative radiotherapy was not feasible. In February 2021, he had a local relapse with zygomatic bone invasion, which was deemed irresectable. Palliative ChT treatment was started (carboplatin AUC 5 plus 5-FU 750 mg/m^2^, every 3 weeks), but progressive disease was seen after four cycles. The patient started second-line palliative cemiplimab treatment in July 2021 and had a clinical and imagiological partial response after 6 months of treatment (Figure 3). 

A complete response was achieved after 24 months, when treatment was discontinued. No immune-related adverse events were reported, and the patient remains disease-free at the time of article writing.

Case three

**Figure 4:**
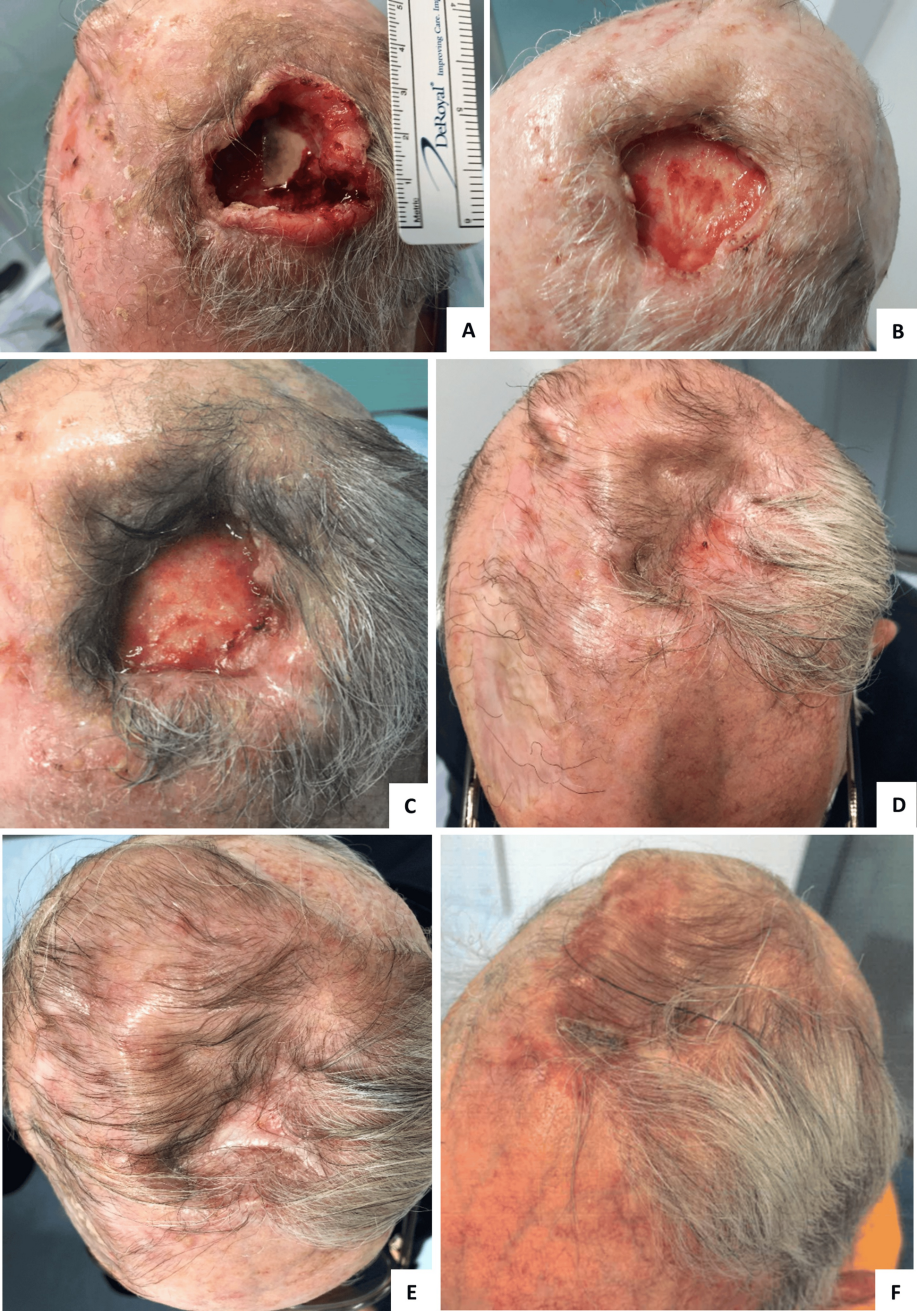
Response to cemiplimab in patient three. A) Patient status before starting cemiplimab (June 2020) - a 3 × 4 cm ulcerated lesion with parietal bone invasion and intracranial extension can be seen. B, C, and D) Photographic documentation 1 month (July 2020), 2 months (August 2020), and 5 months (November 2020) after starting cemiplimab, showing rapid clinical response. E) Complete response was seen in the follow-up appointment of January 2021. F) Sustained complete response 21 months after stopping treatment (March 2024).

An 80-year-old male had a history of arterial hypertension, dyslipidemia, ischemic heart disease, and recurrent CSCC, treated with local excisions between 2016 and 2019. In April 2020, having an ECOG-PS of 1, he presented with a 3 × 4 cm ulcerated right frontoparietal lesion compatible with extensive CSCC relapse (Figure 4A).

**Figure 5:**
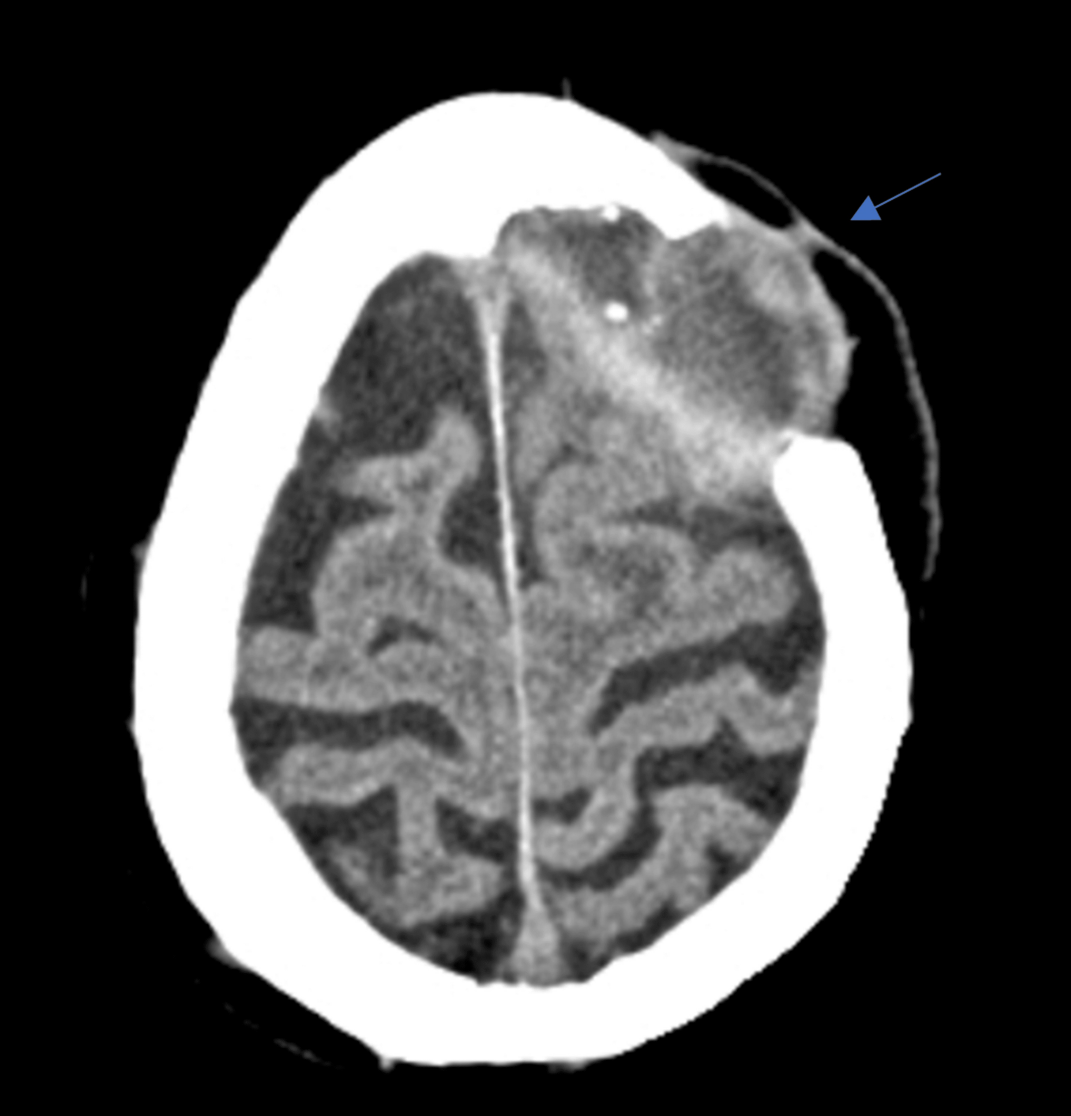
Staging CT scan. CT scan from April 2020 revealing a right frontoparietal lesion with parietal bone invasion and intracranial extension. No distant metastasis was seen.

Staging CT scans revealed no distant metastases, but parietal bone invasion and intracranial extension could be seen (Figure 5). 

**Figure 6:**
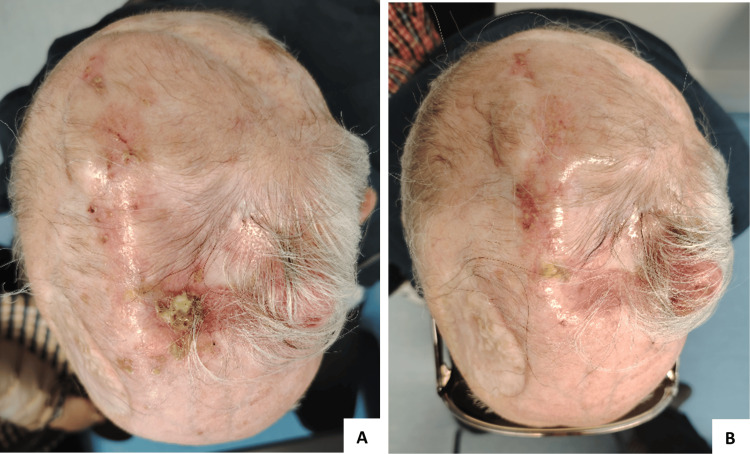
Local relapse of CSCC. A) Patient status before re-starting cemiplimab (October 2024) - a 3 × 2.5 cm soft-tissue mass located in the left parietal area could be seen. B) Stable disease is seen as the best response by the time of article writing, 6 months after cemiplimab rechallenge (January 2025). CSCC: Cutaneous squamous cell carcinoma

The patient started palliative treatment with cemiplimab in June 2020 and had a rapid clinical response (Figures 4B-4F). A complete response was noted by January 2021, and treatment was completed in June 2022, after 35 cycles, with no immune-related adverse events being reported. In October 2024, the patient had a local relapse, as a 3 × 2.5 cm soft-tissue mass was noted on the left parietal area (Figure 6A). Cemiplimab was reintroduced, achieving stable disease as the best response by the time of article writing (Figure 6B).

Case four

**Figure 7:**
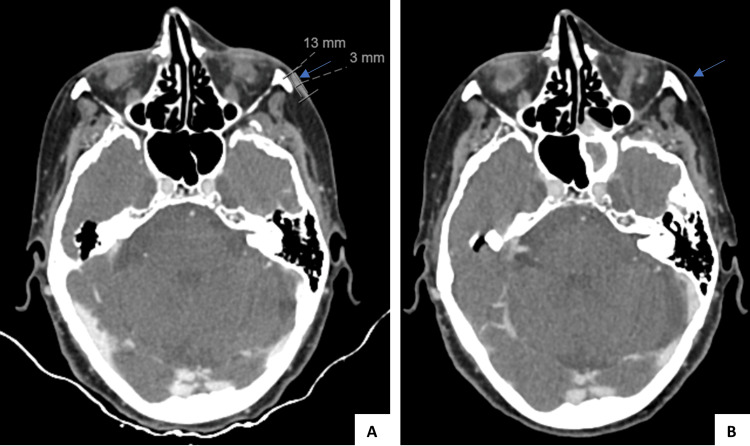
CT-scan images reporting cemiplimab response in patient four. A) Patient status at the start of cemiplimab treatment – a 13x3mm lesion on the left malar region was seen (March 2020), and B) imaging showing sustained complete response 3 years after stopping cemiplimab (February 2025).

A 75-year-old female with a history of arterial hypertension and dyslipidemia had the first left nasal fold CSCC excision in September 2015; in September 2016, after a new excision for a local relapse revealed positive margins, with no possibility of widening, she had local radiotherapy treatment (for a total of 57 Gy). In January 2018, she had another local relapse, which was deemed irresectable, so the patient started ChT-based palliative treatment (carboplatin AUC 5 plus 5-FU 750 mg/m^2^, every 3 weeks, for eight cycles), with stable disease (SD) being the best response. In December 2019, local disease progression was seen - four new lesions were noted: the biggest one on the left malar region with 13 × 3 mm (Figure 7A), and three smaller ones near the upper lip.

**Figure 8:**
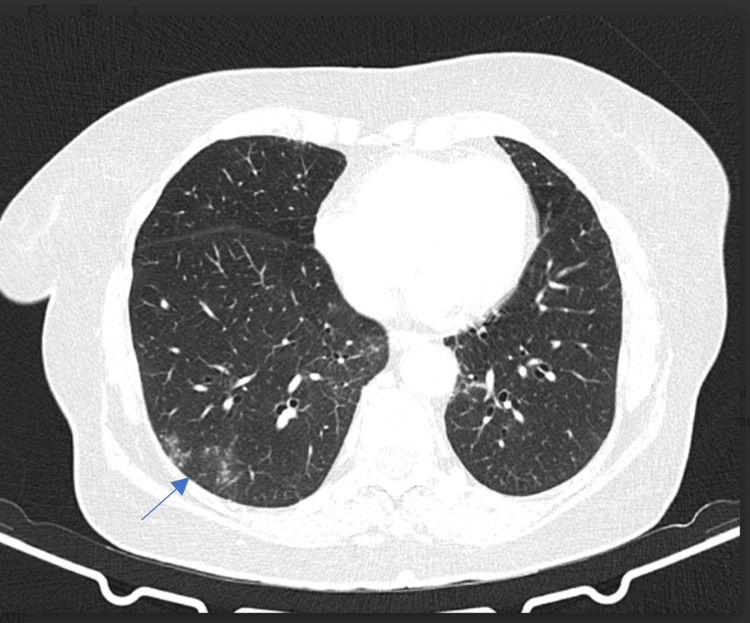
Immune-related pneumonitis. Lung CT-scan showing ground-glass opacities in the right lower lobe, consistent with immune-related pneumonitis grade 1 (CTCAE).

The patient had access to cemiplimab on the basis of an early access program, starting treatment in February 2020. One year after starting treatment, the patient had stable disease on a CT scan, but a de novo 1-cm lesion was noted on the right leg, which was excised and revealed a squamous cell carcinoma in situ. After discussing with the patient, cemiplimab was resumed. In January 2022, CT imaging revealed ground-glass opacities confined to the right lower lobe (Figure 8). 

The patient was asymptomatic, and a diagnosis of immune-related pneumonitis, G1 CTCAE, was made. Treatment was continued, and there was no progression of lung lesions on follow-up CT scans. By March 2022, the patient showed no evidence of disease, and treatment was discontinued. The patient developed G1 CTCAE hypothyroidism, currently supplemented, and maintains a sustained complete response 3 years after stopping treatment (Figure 7B).

Table 1 reveals baseline characteristics and treatment outcomes of the presented patients with advanced CSCC treated with cemiplimab.

**Table 1:** Baseline characteristics and treatment outcomes of presented patients with advanced CSCC treated with cemiplimab. BPH: Benign prostatic hyperplasia; ChT: Chemotherapy; CKD: Chronic kidney disease; CR: Complete response; ECOG-PS: Eastern Cooperative Oncology Group Performance Status; F: female; HTN: Hypertension; IHD = Ischemic heart disease; irAEs: immune-related adverse events; M: Male; RT: Radiotherapy; SD: Stable disease

Patient	Age/Sex	ECOG-PS	Comorbidities	Prior therapy	Cemiplimab duration	Best response (months)	irAEs
1	83/M	1	HTN, hypertensive cardiopathy, CKD, alcoholic cirrhosis	Surgery and RT	27 months	CR (9)	G2 colitis
2	82/M	0	HTN, BPH	Surgery, ChT	24 months	CR (24)	None
3	80/M	1	HTN, dyslipidemia, IHD	Multiple surgeries	24 months + rechallenge	CR (6), SD on rechallenge	None
4	75/F	1	HTN, dyslipidemia	Multiple surgeries, RT, ChT	25 months	CR (24)	G1 pneumonitis, G1 hypothyroidism

## Discussion

Unresectable CSCC can be disfiguring and functionally debilitating. Most of CSCC patients are geriatric, and age-related comorbidities may compromise their tolerance to ChT regimens. Moreover, responses with ChT are low and short-lasting, with median OS typically being less than 12 months [12]. As such, cemiplimab has become the standard of care for advanced CSCC and is capable of providing rapid and durable responses, with a manageable toxicity profile. An update on the results from the EMPOWER-CSCC-1 trial published this year revealed that 13% of patients experienced complete responses, and a median PFS of 22.5 months (7.5 - NE) was achieved. Median duration of response had not been reached at the time of analysis. Moreover, long-term follow-up data presented indicated that complete responses increased over time [13].

This case series represents real-world data showcasing successful and prolonged responses to cemiplimab in patients aged between 75 and 83 years old. Complete responses were achieved in all patients, within 6 to 24 months of treatment. All but one currently maintain sustained responses at 19 months to more than 3 years from treatment discontinuation.

Patient one had cardiovascular comorbidities that deemed him a bad candidate for ChT. After starting cemiplimab, he achieved a complete response within 9 months and is currently disease-free 19 months after stopping treatment. Patient two had a CSCC located in the malar region, which by itself carries a worse prognosis [14]. He did not respond to ChT treatment, but had a partial response after 6 months of cemiplimab treatment, highlighting the limited efficacy of ChT in some of these tumors. Patient three, despite having a deep lesion with intracranial extension and important associated morbidity, had a complete response within only 6 months of treatment. However, more than two years after stopping treatment, a local relapse was seen, for which few treatment options were available, so cemiplimab rechallenge was suggested. Despite it not being standard-of-care, ICI rechallenge may be a viable strategy in selected patients. Limited case-based evidence suggests that retreatment can lead to disease control or re-induction of response, especially in patients with prior durable responses [15]. In fact, the main option for second-line treatment is platinum-based chemotherapy, and some patients, especially the elderly, may not be good candidates. Finally, patient four had had stable disease as the best response to ChT treatment, but achieved a complete response with cemiplimab treatment. Despite the disease control by the one-year mark, she had a metachronous squamous cell carcinoma-in-situ, which was excised. A multidisciplinary decision was made to continue cemiplimab treatment, allowing the patient to achieve a complete response within two years.

Cemiplimab has an acceptable toxicity profile. Two of these patients had immune-related adverse events reported. Patient two had a G2 CTCAE immune-related colitis, which responded to corticotherapy. ICI could have been resumed; however, in this case, having completed more than two years of treatment, a decision to permanently interrupt treatment was made, without compromising the patient’s outcome. Regarding case four, G1 CTCAE hypothyroidism was easily managed with levothyroxine supplementation, and the G1 CTCAE pneumonitis did not worsen with treatment continuation. Notably, three of four patients had cardiovascular disease or risk factors, reflecting real-world populations where comorbidities frequently limit chemotherapy. The favorable safety and efficacy outcomes seen here support cemiplimab use even in high-risk patients traditionally considered unsuitable for systemic therapy.

The decision to stop treatment after sustained complete responses must be discussed with the patients. There is evidence showing that patients who discontinued cemiplimab after achieving a response do not experience significantly different overall survival compared to those who continue treatment, implying that durable responses can persist even after cessation of therapy [16]. This case series supports the decision to suspend cemiplimab in patients with sustained responses.

Limitations of this series include the small sample size, retrospective design, and single-institution setting, which restrict generalizability. In addition, the uniformly complete responses observed may reflect selection bias, since all patients were able to access cemiplimab and complete therapy. These results, while encouraging, should not be generalized to all advanced CSCC patients.

## Conclusions

Cemiplimab offers robust and durable antitumor activity in advanced CSCC, including in elderly patients unfit for ChT treatment. Responses can deepen over time and persist after treatment discontinuation. In this real-world case series, all patients achieved a complete response and maintained prolonged disease control even after treatment discontinuation. Upon relapse, cemiplimab rechallenge may be a viable strategy in selected patients. The safety profile was manageable, and immune-related adverse events, when present, did not preclude long-term benefit. These findings support the use of cemiplimab as a first-line treatment in advanced CSCC and provide real-world evidence favoring treatment discontinuation after sustained responses, with the possibility of cemiplimab rechallenge upon relapse, reinforcing emerging clinical strategies for optimizing immunotherapy duration.
